# Injury reporting in elite ladies Gaelic football and camogie: Perspectives of athlete support personnel

**DOI:** 10.1371/journal.pone.0329679

**Published:** 2025-08-04

**Authors:** Marese Gilhooly, Roisin Cahalan, Kieran O’Sullivan, Catherine Norton

**Affiliations:** 1 School of Allied Health, University of Limerick, Limerick, Ireland; 2 Department of Sport and Health Science, Technological University of the Shannon, Athlone, Ireland; 3 Health Research Institute, University of Limerick, Limerick, Ireland; 4 Physical Activity for Health Research Cluster, Health Research Institute, University of Limerick, Limerick, Ireland; 5 Sports and Human Performance Research Centre, University of Limerick, Limerick, Ireland; 6 Department of Physical Education and Sport Sciences, University of Limerick, Limerick, Ireland; Portugal Football School, Portuguese Football Federation, PORTUGAL

## Abstract

**Objectives:**

This study investigates athlete support personnel’s (ASP) perspectives on injury reporting among elite ladies’ Gaelic football and camogie players. ASP refers to individuals in coaching, management, and allied health and performance related practice.

**Methods:**

A mixed-methods design was employed, comprising an online survey and follow-up interviews. Forty-two ASP, currently or recently (within two years) involved in elite ladies’ Gaelic games, completed the survey. Eighteen ASP subsequently participated in semi-structured interviews. Quantitative survey data were analysed using frequency analysis, while interview transcripts were subjected to framework analysis.

**Results:**

A significant proportion of ASP (43%, n = 18) believe that players do not report all injuries. Most respondents (95%, n = 40) agreed that players often avoid reporting injuries to prevent losing playing time, while 95% (n = 40) cited player fear of being side-lined as a key factor. Furthermore, 93% (n = 39) agreed that players are more likely to report injuries when immediate access to medical personnel, such as team doctors or physiotherapists, is available. Notably, 66% (n = 28) disagreed with the perception that managers view players as weak for reporting injuries, while 19% (n = 8) agreed with this notion. Qualitative findings corroborated these results and identified three overarching themes influencing injury reporting: player factors, system-driven influences, and environmental conditions. Individual factors included a competitive environment that prioritised maintaining team position over health, with experienced players exhibiting greater confidence in reporting injuries compared to younger players, who often perceived management as hierarchical and less approachable. Immediate, consistent access to physiotherapists and other medical professionals was highlighted as a critical enabler of injury reporting. Trust in ASP, built through visible involvement and positive relationships, emerged as a key determinant. Additionally, a supportive team culture that prioritises player well-being alongside performance outcomes was identified as essential for encouraging open injury reporting.

**Conclusion:**

Injury underreporting is a significant issue among elite ladies’ Gaelic games players, driven by personal, system-level, and environmental factors. Of critical importance for teams and organisations is to provide education for players and ASP on the importance and value of injury reporting to support early and appropriate intervention. Concurrently providing improved medical access will help to foster stronger, trust-based relationships between players and ASP, considered crucial for improving injury reporting practices.

## Introduction

Gaelic games are traditional Irish games that include men’s Gaelic football and hurling, governed by the Gaelic Athletic Association (GAA), camogie, the women’s equivalent of hurling, governed by the camogie association and ladies Gaelic football (LGF) governed by the ladies Gaelic football association (LGFA) [1]. LGF is the most played and watched female sport in Ireland and the fastest growing female sport in Europe [2]. Camogie is played worldwide and is one of the most popular female team sports in Ireland [3]. Both LGF and camogie are considered high intensity, multi-directional, field-based team sports with requirements for strength, flexibility, speed and endurance with code specific skills and equipment [4]. Amateurism is central to Gaelic games, yet players can attain an elite intercounty level of participation with time commitments, competition scheduling and performance pathways that are comparable to professional sports [5]. While it is common for intercounty players to hold fulltime jobs or be fulltime students, they can dedicate up to 30-hours per week to training, matches and associated travel and preparation [1]. At the elite level of Gaelic games, while participation remains important, performance has a greater role [6]. To manage their sporting, personal and professional commitments and demands, players are supported by a dedicated team of professionals [6]. The athlete support personnel (ASP) network of individuals includes the manager, medical personnel, strength and conditioning (S&C) practitioners and technical staff [7]. ASP are well-positioned to manage athletes’ physical and psychological health, to enhance performance and minimise injury risk [7]. They are often the frontline contact with whom the athlete interacts when injured [8].

While there are many health benefits to sport participation, it does involve a risk of injury [9]. The high physical demands of ladies Gaelic games likewise leads to injury, with an injury incidence in elite camogie and collegiate LGF [10,11], comparable to female elite team sports including soccer, rugby sevens and field hockey [12–14]. To guide the effective implementation of specific injury prevention measures, injury surveillance (IS) must first establish the extent and nature of sports injuries [15]. Yet divergent perspectives among players, coaches and medical personnel on the value and importance of IS have been highlighted [16]. When resources are limited, other needs can be seen as more critical [16].

From a player perspective in elite LGF and camogie, injuries are not reported for reasons including individual player mindset, a fear of missing playing time, lack of accessible and available medical personnel and a perceived team culture where injury reporting is viewed negatively and/or not encouraged [8]. More than a third of injured elite men’s Gaelic footballers played matches despite their injuries while a majority reported being responsible for the final decision to participate when injured [17]. The influence of ASP can have a powerful impact on the injury reporting behaviours of athletes [8]. A key factor in the injury reporting behaviour of high-school athletes was the relationship with the athletic therapist (AT) [18]. Access to ATs facilitated more timely recognition and treatment of concussion in collegiate athletes [19]. Coaches can underestimate the frequency of injury underreporting, suggesting a disconnect between actual and perceived injury reporting behaviour [20]. Open communication and frequent interactions with ASP can encourage injury reporting [21], though difficulties communicating with managers can discourage players from reporting [22]. A collaborative ASP network can enhance individual, team and organisation achievement [23] and has been deemed a crucial component in monitoring athletes through both quantifiable measures and open communication that can gather information not captured by an objective test [24].

To have a clearer understanding of the injury context and to assist in the design of user friendly and effective injury surveillance systems (ISS), the perspectives of players and ASP need to be explored [16]. At elite level of ladies Gaelic games, previous research to date has mainly relied on player self-reports [8,25], while no research has been undertaken to investigate the perspectives of ASP. As the main end-users of ISS and injury prevention measures, it is important to understand the perspectives of athletes and ASP on injury and reporting [26]. The views of ASP are not frequently represented in sports injury prevention studies [27]. Understanding their views regarding injury reporting in elite LGF and camogie is vital for developing context specific evidence-informed injury reporting, prevention and management strategies. This understanding could provide new and broader insights to encourage injury reporting and optimise health and well-being in these sports.

Therefore, this study aims to investigate the perspectives of ASP on the injury reporting practices of elite ladies Gaelic games players.

## Methods

### Study design and ethics

This mixed methods research study adopts a pragmatic philosophy recognising that social, historical and political contexts influence the scientific process with knowledge construction occurring through conversations and exchanges between and within communities of people [28]. Pragmatism accepts that there are multiple realities that are grounded in the environment, only encountered through human experience and based on beliefs and habits that are socially constructed [29]. Pragmatism and mixed methods research supports the concept that by moving back and forth between data sets, the knowledge produced by each one can give a more holistic, nuanced and deeper understanding and insight of the scientific inquiry [30]. This study used a sequential explanatory design with quantitative data collected with a survey to evaluate the perspectives of ASP on the injury reporting behaviours of ladies Gaelic games players followed by qualitative data collection in interviews to gain deeper insights and explanations of these perspectives [31]. The quantitative phase was designated as the priority phase and was followed by the qualitative phase with data analysis carried out separately before amalgamating the quantitative and qualitative data. Ethics approval was granted in writing by the research ethics committee of the Faculty of Education and Health Sciences at the University of Limerick (2024_04_03_EHS). Written informed consent was provided by all participants. The processing of data was carried out in accordance with the General Data Protection Regulation (GDPR)/Data Protection Acts 1988–2018 (‘Data Protection Law’) and in accordance with the university’s Research Privacy Notice. Each survey and interview respondent were given an alphanumeric code to maintain pseudonymity and confidentiality. The collected data were kept private and stored securely and safely on the principal researcher’s (MG) password protected computer, on the researcher’s university approved shared data storage repository One Drive.

### Data collection

#### Quantitative data collection.

A cross-sectional survey (S1 File) was designed to investigate the perspectives of the ASP on the injury reporting practices of elite adult LGF and camogie players. The disseminated survey and data generated for this paper were completed using Qualtrics software, (Qualtrics https://www.qualtrics). The survey was open to responses from August 12 to November 30 2024. The survey was developed based on previous injury epidemiology research including concussion reporting behaviours of high school athletes, experience of pain and injury in collegiate athletes and injury surveillance practices in competitive swimming and elite ladies Gaelic games [8,19,32–34]. The survey was comprised of three main sections. The breakdown of the survey is displayed in S1 File. Section one consisted of the study details, ethics and consent. Section two consisted of nine demographic questions, with pre-defined answers supplemented by open text box answers to provide greater depth, which addressed participants’ support role in elite LGF or camogie, the sport code, the team playing level, the most recent role within the team, number of years working within the elite level and educational and professional accreditations. Section three consisted of twenty statements that explored participants’ perceptions of reporting practices and behaviours of athletes. Participants rated their level of agreement with each statement using a Likert scale from 1 = strongly agree to 5 = strongly disagree. These statements were arranged in three subsections. The first consisted of ten statements related to the player, the second of six statements related to the organisation and the third subsection included four statements related to the team culture. Face validity was conducted in consultation with a panel of five ASP with extensive experience working in ladies Gaelic games. These ASP were required to review and provide feedback on the survey in terms of clarity, relevance and suitability for the target audience. Thereafter, amendments were made to the survey in line with these consultations in the form of clarity and consistency in phrasing of the questions and enhanced technical terminology. Specifically: Question 5, ‘What is/was the level of the team?’, the level was changed from division to championship tier level; Question 6, ‘What is/was your most recent support role within elite ladies Gaelic football or camogie?’, the ‘Sports Scientist’ role was removed as it was deemed to be unspecific for a precise ASP role; Question 11, ‘Please specify the professional accreditations you hold’, was worded to align with the best practice recommendations presented in the Gaelic Games Player Pathway [6]. Subsequently the survey was pilot tested, between 27/05/2024 and 10/06/2024, on three purposively selected ASP with experience working in club level ladies Gaelic games. No additional changes were made after the pilot. The pilot data were not included in the final analysis. Those who part took in the pilot study did not participate in the final study.

#### Qualitative data collection.

A semi-structured interview guide (S2 File) was developed by the authors based on previous literature pertaining to injury prevention and management in soccer, specifically research investigating (i) perceptions on injury and reporting and (ii) engagement of soccer players, coaches and medical team, with a context specific ISS [35,36]. As the research team had recently conducted qualitative research investigating perspectives on injury reporting of elite ladies Gaelic games players [8], a pilot study was considered unnecessary. Semi-structured interviews were conducted on-line via Microsoft Teams (Version 1808) between September 09 and November 18 2024. All interviews were conducted by the first author. In the semi-structured interviews participants were first given information on the first author’s professional background, confidentiality and the voluntary nature of their participation was explained. The interview discussion then began aimed at establishing participants’ understanding of injury reporting behaviours of elite ladies Gaelic games players and then explored measures and strategies that could enhance reporting. Interview questions (S2 File) included open ended questions including, ‘Do players report all injuries?’. While the interview guide provided structure and allowed the conversation to evolve, a flexible approach using elaboration and clarification probes such as ‘Can you explain why you think that is?’ and ‘Can you expand on this?’, facilitated discussion beyond the interview guide ensuring consistent depth throughout the interviews.

### Participants and recruitment

Purposive and snowball sampling from the authors’ professional and Gaelic games network of contacts were used to recruit ASP, who were currently or within the previous 2-years involved in a support role in elite LGF or camogie. To minimise selection bias and ensure broad representation in support role, team level, geographical region and sport code, recruitment strategies including purposive and snowball sampling, recruitment through the two national governing bodies (NGBs) and the authors’ national professional bodies, were employed. All participants who completed the survey were invited to participate in the qualitative element of the study. The recruitment process was continual until saturation of information was achieved [37]. The adequacy of the collected data to address the research question was reviewed during the data collection and analysis process [38]. Ongoing review of participant responses and refinement of themes in concert with the broader research team, affirmed the collected data captured diversity and nuances providing significant patterns of shared meaning [39] with variation across participants including specific support roles and team level. The role of manager, in both LGF and camogie, was deemed to be underrepresented with only two managers from a cohort of fifteen. Further targeted purposive sampling was employed from the authors’ professional and Gaelic games network of contacts, to specifically recruit participants currently or within the previous 2-years involved in this role.

### Data analysis

#### Quantitative data analysis.

All responses to the injury reporting behaviours survey were transferred to Microsoft Excel Version 1808 and SPSS Version 23 (IBM Corp, USA) for further analysis. Descriptive statistics were calculated in SPSS. Frequency analysis was used to analyse the quantitative responses with data presented as absolute frequencies and percentages.

#### Qualitative data analysis.

Data was transcribed verbatim with pseudonyms assigned to each participant to retain anonymity. Interview recordings were deleted once transcription was complete. Data was then transferred to a password protected Microsoft Excel spreadsheet for analysis [40]. A framework approach (FA) was used to underpin data analysis [41], involving a five-step process of 1) data familiarisation; 2) identifying a thematic framework; 3) indexing; 4) charting; 5) mapping and interpretation. As FA is considered theoretically flexible it can be adapted for inductive or deductive analysis or a combination of the two [42]. The a priori themes (and subthemes) of the player (subthemes: timing and nature of injury, demographics, financial means, individual mindset), the organisation (subthemes: access to medical support personnel, relationships, reporting structures) and the environment (subthemes: club culture, approach of management), were preselected based on previous research investigating elite ladies Gaelic games players’ perspectives on injury reporting [8], while emerging and analytical themes and subthemes in the data set were recognised ensuring all aspects of the data were captured [42]. A key characteristic of FA is the development of a data reduction framework with themes forming the basis for this framework [43]. It is an iterative process, with the interconnected non-linear stages providing the scaffold that guides the analysis [44], and aligns well to pragmatism with its reflective and analytical approach to scientific inquiry [28]. As data analysis progressed, reviews and interpretations of the data were collaboratively and reflexively conducted by all authors. Open discussions continued until agreement was reached on the final interpretations of the data. There were no instances where consensus was not reached. This process enhanced consensus on the development, refinement and defining of codes and themes and on determining data saturation. Data saturation was considered achieved when no new themes, insights, or perspectives emerged during successive interview discussions, indicating that additional data collection was unlikely to yield further meaningful information.

### Trustworthiness and rigour

All authors were involved in the analytical process to review initial and final themes and subthemes. This ensured a greater understanding of the research topic as final themes and subthemes were agreed upon. The main author (MG) is a female Chartered physiotherapist and PhD student who has many years of pitch-side and clinical experience working within Gaelic games and other sports of various levels. All co-authors are senior and experienced academics and researchers who have published widely on injury surveillance in elite sports and either currently (CN) or previously (KO’S) have worked as ASP in intercountry/elite Gaelic games. While it is acknowledged that the analytical process is subjective and open to different interpretations, collaboration allows researchers to pool and organise meaning and assumptions in the data [45]. This study adhered to the standards for reporting qualitative research [46] to ensure rigour during the FA process (S1 Table). Member checking ensured credibility of the collected data with no changes subsequently made. The main author-maintained field notes to record observations and to allow reflection throughout the data collection and analysis process.

## Results

Forty-two ASP participated in the survey. Of these 42, 18 also participated in the semi-structured interviews that lasted 42 ± 8.4 (mean±SD) minutes. Roles of participating ASP are reported in Table 1. The team level and academic/professional profile for each participant was collected. Additionally, the specific team was collected for each interview participant. However, the ladies Gaelic games community in Ireland is relatively small with familiarity between and across teams. Therefore, to ensure anonymity of all participants, the detailed background information of the survey participants only is presented at group level in Table 2. The interview participants’ individual ASP role and sport code is presented in Table 3. To avoid compromising anonymity, the basic geographical distribution of teams is reported based on each team’s provincial location [47]. Fifty percent (n = 9) of teams were located in Leinster, 22% (n = 4) in Munster, 17% (n = 3) in Connaught and 11% (n = 2) located in Ulster.

**Table 1:** Participants’ role within athlete support.

Role	Survey respondents N = 42	Interview respondents N = 18
Manager	8 (19%)	5 (28%)
Coach	6 (14%)	2 (11%)
Physiotherapist	10 (24%)	4 (22%)
Athletic Therapist	6 (14%)	2 (11%)
Strength and conditioning coach	5 (12%)	3 (17%)
Nutritionist	4 (10%)	1 (5%)
Psychologist	3 (7%)	1 (5%)

Results are presented as frequencies and percentages (%).

**Table 2:** Survey participants’ Gaelic games, academic and professional profile.

Characteristic	Frequency (%)
Sport code	LGF 32 (76%) Camogie 8 (19%) Both 2 (5%)
Level of the team	Senior 26 (62%) Intermediate 10 (24%) Junior 16 (14%)
Years working in intercounty setting	<1 year 3 (7%) 1-2 years 19 (45%) 3-5 years 13 (31%) 6-10 years 4 (10%) >10 years 3 (7%)
Highest academic qualification	No formal academic qualification 1 (2%) Bachelor’s degree 17 (41%) Master’s degree 22 (52%) PhD 2 (5%)
Professional accreditation (related to ASP role)	No professional accreditation 6 (14%) Clinical accreditation 21 (50%) Performance analysis accreditation 1 (2%) Coaching accreditation 14 (33%)

**Legend:** LGF = ladies Gaelic football; senior level = teams competing in the premier first-tier championship competition organised by the ladies Gaelic football association or camogie association; intermediate level = teams competing in the second-tier championship competition; junior level = teams competing in the third-tier championship competition.

**Table 3:** Interview participants’ ASP role and sport code.

Pseudonym (n = 18)	ASP role	Sport code
**Lee**	Chartered physiotherapist	LGF
**Jamie**	Manager	Camogie
**Robin**	Sports psychologist	LGF
**Mike**	Strength and conditioning coach	LFG
**Dillon**	Chartered physiotherapist	Camogie
**Daire**	Strength and conditioning coach	LGF
**Cory**	Chartered physiotherapist	LGF
**Alex**	Nutritionist	Camogie
**Jo**	Coach	LGF/camogie
**Charlie**	Athletic therapist	LGF
**Ryan**	Strength and conditioning coach	LGF
**Sam**	Chartered physiotherapist	LGF
**Billie**	Manager	LGF
**Jed**	Manager	LGF
**Shay**	Manager	LGF
**Eddie**	Athletic therapist	Camogie
**Andy**	Manager	LGF
**Terry**	Coach	LGF

### Quantitative results

**Fig 1:**
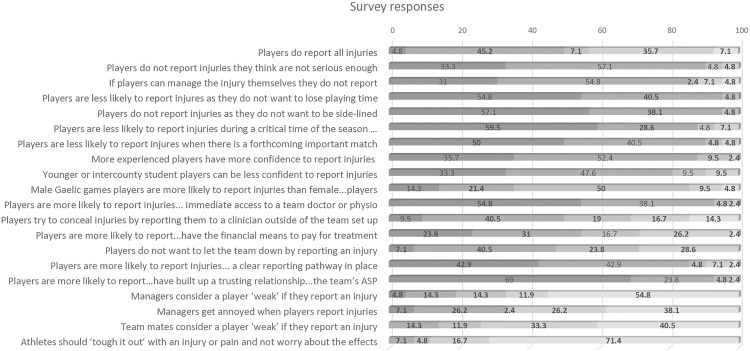
Survey participants’ responses. The bar chart visualises the percentages (%) of responses for each factor related to the player, the organisation, and team culture. The colour intensity represents the percentage of respondents who selected each option, with darker colours indicating agreement. Legend: ASP = Athlete Support Personnel, Physio = Physiotherapist.

Quantitative results pertaining to sections exploring player, organisational and cultural factors are outlined here with further detail provided in Fig 1 (S2 Table).

#### Factors related to the player.

A large majority (90.4%, n = 38) of ASP agreed that players were less likely to report injuries perceived as less serious. Overall, ASP (42.8%, n = 18) disagreed that players report all injuries. A large proportion (85.8%, n = 33) of participants agreed that players are less likely to report injury if they feel they can manage the situation themselves. Participants agreed that factors associated with a reluctance to report included to avoid losing playing time (95.3%, n = 40), or being side-lined (95.2%, n = 40). Eighty-eight percent (n = 37) and 90.8% (n = 38) of participants respectively, agreed that players were less likely to report injuries during a critical time of the season, or if there was a forthcoming important match. Most ASP agreed (88.1%. n = 37) that more experienced players have more confidence to report injuries, while 80.9% (n = 34) agreed that younger or intercounty student players are less confident to report injuries. A greater proportion (35.7%, n = 15) of participants agreed that male Gaelic games players are more likely to report injures compared to females, while 14.3% (n = 6) disagreed, and 50% (n = 21) neither agreed or disagreed.

#### Factors related to the organisation.

A large proportion (92.9%, n = 39) of ASP concurred that immediate access to a team doctor or physiotherapist may encourage players to report injuries. Fifty percent (n = 21) of respondents reported that players may try to conceal injuries by reporting them to a clinician outside of the team set-up, compared to 31% (n = 10) who disagreed. Participants (54.8%, n = 23) agreed that players are more likely to report injuries when they have the financial means to pay for treatment compared to 28.6% (n = 12) who disagreed. Almost half (47.6%, n = 20) of participants agreed that players do not want to let the team down by reporting an injury, while 85.8% (n = 36) agreed that a clear reporting pathway facilitates better injury reporting. An overwhelming 92.8% (n = 39) of participants agreed that players are more likely to report injuries if they have established a trusting relationship with the team’s ASP.

#### Factors related to team culture.

Sixty-six percent (n = 28) of participants did not agree that managers would consider a player weak if they reported an injury while 19.1% (n = 8) agreed. Yet 33.3% (n = 27) agreed that managers get annoyed when players report injuries, while 64.3% (n = 27) disagreed. Participants (78.8%, n = 31)) disagreed that teammates would consider a player ‘weak’ if they report an injury. Overwhelmingly 88.1% (n = 37) of ASP disagreed that players should ‘tough it out’ with an injury or pain and not worry about the effects.

### Qualitative results

**Fig 2:**
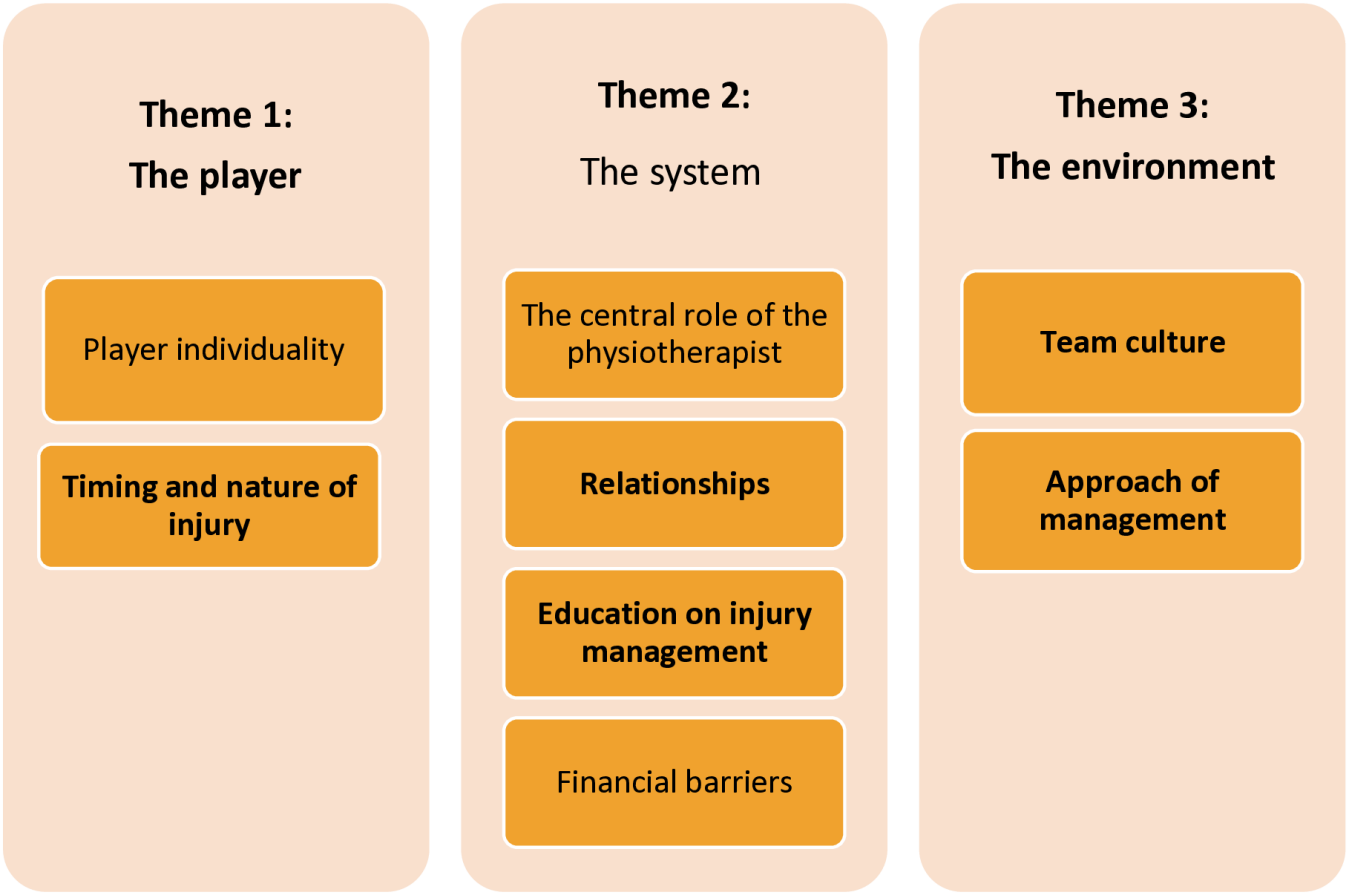
Thematic diagram illustrating a priori and emergent themes and subthemes.

The emergent theme, the system, was deemed more appropriate than the a priori theme, the organisation, in the context of perspectives of ASP. Therefore, the themes of the player, the system and the environment were used to frame the findings underpinned by a priori and emergent subthemes. Themes and subthemes of the interviews are presented in Fig 2. While the themes and subthemes are described separately, interaction between them can inform the narrative of the ASP perspectives on injury reporting [36].

#### The player.

***Player individuality*:** Participants noted that more experienced players have greater injury awareness and confidence to report injuries than younger, less experienced players, who feel a sense of duty to continue playing. As Daire, a S&C coach working in LGF, stated, ‘more established players are more secure in their position … have been around the system, around the team a lot longer … they’re more comfortable communicating those injuries…’. More context is provided by Mike, a LGF S&C coach who stated, ‘… even with a bit of a niggle or an injury (players) may be able to perform better on the field than some of the less experienced…less able players’. While Jed, a LGF manager reported, ‘(the more experienced players know) it’ll only … get worse and even if they do play with injury, they know it’s actually nearly worse than not playing’. Eddie, an AT working in camogie stated, ‘the older players will actually come to you … (and say) this is an issue, just wanna get it flagged…the younger players probably think it’s nothing… I’ll run it off …older players probably just have more knowledge...the younger players probably think … I’m going to be out for X amount of games, I’m not going to get it checked out’.

In contrast, some younger players perceived a power dynamic, particularly if they feel less certain about their position in the team. Billie, a manager in LGF stated, ‘… (younger players are) worried about maybe if they don’t train, they don’t play’, while Charlie, an AT working in LGF described how, ’the younger girls found it very hard to actually like speak to myself or management’. Lee, a Chartered physiotherapist working in LGF discussed that, ‘they (younger players) feel that they might be judged if…they’re injured or they’re seen to be injury prone...managers might be like,…what’s going on... They’re always injured, is it in their head’. Ladies Gaelic football Manager Jed added, ‘the younger the player... the less likely they are to record injuries even more so I suppose the less experienced the player, the less likely they are to actually tell you about injury’. The importance of playing intercounty Gaelic games, particularly in the lives of younger players, was described by Eddie, an AT working in camogie, who stated that, ‘…playing camogie at that (intercounty) level is a big thing for (younger players)... part of their identity... take that away through injury... it sort of leaves them with a piece of them missing... can be quite isolating for them......Older players tend to … be not as identity restricted’.

Participants reported that players are less likely to report injury to avoid losing playing time or being side-lined. It was reported that players at the intercounty level try to avoid jeopardising their position on the starting team due to the high level of competition. This was discussed by Jamie, a camogie manager, who stated, ‘it’s so competitive and if a team is going well, I suppose players don’t want to lose their place’ and by Mike, ‘they want to be on that first fifteen… starting team… they don’t want someone else coming in to try and take that spot’. Robin, a sports psychologist working in LGF described how, ‘(players feel) I need to train to prove that I deserve my place on the team…I don’t want a fuss made...a lot of them put pressure on themselves … I need to keep going here to prove myself’. Strength and conditioning coach Daire added, ‘they (players) don’t want to miss out or to be overlooked… to get onto the team...’. In addition, as S&C coach Ryan stated, ‘players are working hard to get to that (elite) level...they’d be afraid of the consequences (of reporting) in terms of being dropped, being isolated, being put on the side-line or being put off the team, being dropped off the squad’. Football manager Shay elaborated further, ‘there’s a touch of bravado…I’m not injured, I’m OK, I’m tough … they’re worried about, you’re gonna lose your place on the team…not gonna get game time…not gonna start, so I’ll hide it (the injury)’. He described the competitive environment of the intercounty level, reporting how, ‘people are competing for places … if I’m injured, I’m going to go back to the bottom...of the heap, so I’ll play through it (injury)’.

Yet it was acknowledged that failure to report can have consequences for both individuals and the team. Dillon, a Chartered physiotherapist working in camogie stated, ‘they don’t know that it might get worse… there’s a lack of understanding there’, with LGF manager Billie reporting, ’they’re not being a team player by going out in the pitch and not being able to perform at their maximum… they’re not actually doing the team any favour’. Daire added perspective describing that, ’if players were flagging even what they perceive as minor issues…that can…give us a warning sign … we can modify training load … try and mitigate some of this injury risk’. He further elaborated on the importance and perceived consequences of flagging minor issues, ‘to add context to maybe when they (players) perceive the management might think that they have a dip in performance…to provide that context …why I was playing poorly tonight in training…because of this injury, so … management is aware… they don’t want to miss training… (they have a fear that) if they’re reporting…they can’t train tonight…fear they will be viewed negatively by the coaches’.

The fear factor of losing a place on the team was reported with an acknowledgement that this can be a natural and inevitable outcome of being injured. However, having a pathway back for players was acknowledged as providing reassurances to players returning to selection. Jed stated, ‘They think the injury is going to last for weeks and they’re going to lose their place and they’re never going to get their place back’. Camogie manager Jamie discussed how,’ (in elite sport) … if you do miss a couple of games and someone comes into your position …there’s no guarantee you’re going to get your position back’. Jamie explained further, ‘if a really experienced player gets an injury … they’ve played for two or three years, so you know exactly what they’re capable of … they’re far more likely (once recovered) to get (back) into the team’. Billie shared that, ’we’re all working towards getting players back on the pitch and when the players … see a pathway…that constant communication… the injury situation just ties in with that they know that they can come to you (if they are injured)’.

Issues regarding a lack of supports for female athletes were discussed. Participants reported that if players are unfamiliar with receiving support, they may struggle to make use of it when it is made available. As clearly stated by Alex, a nutritionist working in camogie, ‘I often feel that women are nearly tougher…in a good way and in a bad way…they will put up with an awful lot more … they’re not used to...being supported’. Coach Jo described how,’at club level it’s really poor (the support for female players)... you could have one person on the line at club level with a physiotherapy bag and he or she is the manager, the coach, the S&C and the physiotherapist...it’s just not good enough. …the clubs and the county boards need to do better to support females’. This was further expressed by Chartered physiotherapist, Cory, who stated that, ‘particularly in female intercounty set-ups, these girls are coming from clubs where there wouldn’t be a physiotherapist sometimes not even at matches so they’re used to trying to self-manage’.

***Timing and nature of injury*:** In addition to individual player factors, participants discussed injury-related factors that affected reporting. Contrasting views on the reporting of injuries at critical times of the season were presented. As stated by S&C coach Daire, ‘in the build-up to big matches … close to championship … there seems to be never any injuries … (players) don’t want to be overlooked for selection’. Similarly Chartered physiotherapist Cory described how, ‘if you’re coming up to a really big competitive match, (players) would be worried… (the physiotherapist) won’t let me train Friday night and then I’m out of running for the match on Sunday’. Yet the experience of Sam, a Chartered physiotherapist working in LGF was that, ‘…they’re definitely more alert to injuries and they want to be 110% … they want to manage it as best they can before a big match like it’s always a busy time’.

Participants stated that if the injury was not visibly affecting performance, was considered minor or the players felt they could manage the injury themselves, they can be less likely to report it. However, it was reported that players are more likely to come forward when the injury becomes unmanageable. Chartered physiotherapist Lee described that, ’if you’re moving relatively OK and no one’s really gonna notice (less likely to report)’, while manager Billie reported, ‘if they feel the injury isn’t … going to affect … their performance (not as inclined to report)’. More context was provided by S&C coach Mike, who stated, ‘if it’s a minor injury, they tend to try to play on and maybe disguise it a little bit or deal with it themselves or play through it…’. Coach Jo added that, ‘(players can) genuinely feel that they can work through it… they know their bodies better than any of us’, while AT Charlie stated, ’once it gets to a point where they can’t conceal it, then they kind of come more readily to you’.

It was discussed that there is a realisation that playing through injury is not always sustainable and that players can learn the implications of not reporting through experience. Dillon stated, ‘(players realise) this is not a manageable injury in the way that you’re currently managing it’, while sports psychologist Robin described how, ‘if you do play on...things don’t necessarily get better...they didn’t report an injury previously and they learned the hard way’. However, if players have experience of an injury and being side-lined, they can be reluctant to report as Chartered physiotherapist Cory reported, ‘a player that has been through this before...been out for six to eight weeks... knows what this feels like…knows what it means...don’t want to go through that again (being out)’.

#### The system.

While player- and injury-specific factors were discussed, broader system level factors were described.

***The central role of the physiotherapist*:** The important role of physiotherapy from an injury reporting perspective was discussed. Camogie manager Jamie stated, ‘physiotherapy should be part of their day to day…the first thing they should think of from an injury point of view is, I’m going to report this to the physiotherapist…’. There was broad consensus on this as LGF manager Shay and sports psychologist Robin respectively, agreed that, ‘we would have a Chartered physiotherapist available at every session…that would be the first port of call (when a player sustains an injury) …absolutely crucial (to have a physiotherapist) …couldn’t do without him, shouldn’t do without him’, and’...a physiotherapist at every training session… that’s probably the big thing … the one resource that is needed at every session… to support them if there is an injury’. This was reiterated by S&C coach Mike and manager Jed respectively who added that, ‘the physiotherapist is always there more than an hour before training to meet any players’, therefore, ‘(as) the physiotherapist is more engaged with the players, players are obviously going to trust them more’.

***Relationships*:** Additional significant contributions from medical personnel specifically the physiotherapist were described further.

The time spent by the physiotherapist with the players and the need for ASP to be an integral and visible presence within the team structure was reported. Terry, a LGF manager stated, ‘The physiotherapist spends more time with the players … than the management team does’ while Jamie described how, ‘physiotherapist, one of the hardest jobs at intercounty, they’re probably first there, last to leave…’. Furthermore, nutritionist Alex reported, ‘we generally work tight together…talking to each other… you need to be present … to get to know everybody… you’re compromising player welfare if you’re only dipping in, dipping out… dipping in, dipping out might be the only budget’.

It was further discussed by participants that the experience and credentials of the ASP helped to foster trust and encourage injury disclosure. As Alex stated,’…when they know you’ve experience… there’s more… respect…trust…we can talk… there’s an extra worry on the player when they’re not dealing with someone who is very specialised in the sporting side of things’. Coach Jo explained that, ‘there does need to be a Chartered physiotherapist...players know this is the avenue I go and report to… a qualified S&C coach your bachelor’s, your master’s it’s a prerequisite… a qualified coach… (there are) a lot of coaches coaching in the intercounty game who haven’t even sat a formal (coaching) education course’. Managers Sam and Jed respectively stated that, ‘making sure that the medical staff…physiotherapy staff … are well trained... have sporting knowledge... so they (players) can trust the physiotherapist or the GP... they are well equipped to deal with their injuries or any health concerns’, and ‘reporting of injuries was far more likely …(if) the physiotherapist or therapist … was … experienced … they were able to get to the bottom of the injury… (players) trust the person and their knowledge’.

Maintaining specific standards within the ASP was stated to reassure players. Manager Billie clearly acknowledged that, ‘…they (players) can see through a bluffer… that level of expertise has to be there’, while Chartered physiotherapist Dillon stated, ‘(players) trusted that we are sports physiotherapists... we had a better understanding of how to manage things to keep you playing…We weren’t just pulling players…Nutritionist Alex added that, ‘when you have experience … we can talk nearly woman to woman as opposed to practitioner to athlete’.

***Financial barriers*:** Other factors impacting willingness to report were also discussed.

It was reported that if the fee for external/private physiotherapy was covered without need for upfront payment it would facilitate reporting. Dillon reported, ‘that was one more barrier taken away… big relief…that’s probably a facilitator...for them to be a little bit more open...it’s not a financial burden on them’. While AT Charlie stated, ‘(players) have to go through a process to apply to get some money from the …injury fund … that kind of puts them off reporting injury…they (governing bodies) make it quite difficult for girls to get support... they have to pay out of their own pocket first… that’s definitely one obstacle that could be taken out...’, and manager Jed reported that, ‘if they know they’re going to get dealt with without a cost on them … they’re more likely to do it (report)’.

A lack of awareness and the complexity of the application and reimbursement process of the governing bodies’ injury schemes, specifically the LGFA injury fund, was reported. Jed stated, ‘… they (players) don’t even know the injury fund exists...I don’t think there’s enough about the injury fund… all the social medias…all my emails …I have not seen the injury fund mentioned’. Similarly, sports psychologist Robin described how, ‘a lot of the players … they’re not educated about the injury fund … don’t know how to access it’. Ryan, a S&C coach in LGF reported that, ‘(it’s) a very difficult process…a long-haul process you’re missing games, championship time… they’ll end up being out of pocket as opposed to go through the whole rigmarole… downloading the application, blah blah blah’. This sentiment was reciprocated by Eddie, an AT working in camogie who stated, ‘it’s too cumbersome…time consuming…paper top heavy...very easily streamline this…make it very easy… what happens is…I’m just gonna pay…I can’t be bothered with the hassle...’.

***Education on injury management*:** While the financial burden on players can be significant, other equally important factors impacting injury reporting were discussed.

The importance of education about injuries and reporting, making players aware of the ASP and supports available to them to encourage proactive engagement was acknowledged. Manager Jamie stated, ‘education…the players have to understand that … if you don’t report an injury to physiotherapist … you could end up with a serious problem … early prevention and early treatment …’. Chartered physiotherapist Dillon added, ‘from a general injury perspective… educating players around what actually is an injury … it mightn’t even mean …stopping training … (or having) to step out of play…the intervention can be small … they perceive that we’re going to make a big deal of it…you can’t train for six weeks… they just don’t have that baseline education’. Cory, a Chartered physiotherapist working in LGF explained that, ‘it’s just promoting the girls to engage for their own welfare and for their own good... it’s about...these people are here in a professional capacity to support and to help you but if you don’t come to them, they can’t help you’. Chartered physiotherapist Sam and manager Andy, respectively highlighted the, ‘lack of knowledge or awareness around…what could …(be) classed as an injury’ and ‘more awareness of … type of injury …more education as their season is going on’.

#### The environment.

Additional to system level factors, the broader context surrounding elite ladies Gaelic games players was also explored.

***Approach of management*:** It was reported that management’s reaction to injury can be challenging and inconsistent as S&C coach Daire stated, ‘conflicting messaging from (stakeholders)… the physiotherapist, the manager and the S&C voicing the need to address … injuries …the selectors might have different attitudes and beliefs … just get on with it and to be a bit tougher’. Coach Jo reported that, ‘most managers … follow up their actions … mean well for the players … will listen to educators, medical teams… once it comes into championship, they may have a different agenda’, while Chartered physiotherapist Lee explained that, ‘the want to win…can override that (player welfare)’. Another perspective was given by S&C coach Ryan, ‘it depends again on the educational status…the interest of the coaches…science or traditional based … more traditional…can be very raw just get on with it, wrap it up there, get out to hell say nothing’. Coach Jo added, ‘it comes back to … building relationships … your people skills, it’s not how much you know, it’s how much you care... if they know I care … they’ll probably trust me, they’ll probably lean on me that little bit more’.

Additionally, the important role of a supportive management structure was discussed. Manager Jed stated that, ‘(management) would always be there to kinda help and support the players…that’s a happy player. A happy player is a good player…a happy camp is a good camp and we work hard on that’. This is in contrast to a more hostile environment, as nutritionist Alex reported that, ‘toxicity can creep in…people will bond through negativity …players are clever… players know the vibe, they know what’s what’. Chartered physiotherapist Lee described how, ‘the manager at the start of the year … just saying it like, (injury) it happens everyone just please let us know if you’re hurt…there’ll be no judgment…(players) might feel more comfortable reporting’.

***Team culture*:** A mutual and shared responsibility from players and ASP to create an open and communicative culture was discussed. Furthermore, it was reported that if players know that they can openly seek support, it can encourage reporting. Manager Shay explained, ‘we can find a work around for every problem … we need to know what the problem is first … you have to tell me…if I create this environment you have to buy into that environment… if I’m being open and commutative with you, you have to be open and communicative back with me’. Similarly, S&C coach Mike stated, ’that would have been an important thing for … the whole management team to make sure that we fostered an environment, that the door is always open, and you can always call’. Charlie added that, ‘within our set up it was definitely encouraged to be open and honest about … injuries... it makes it easier for everybody’.

The role of players and management individually and cohesively in creating the team culture was elaborated upon. Andy, a manager working in LGF stated, ‘a player is a player and there is a selfishness in a player that they want to play all the time… we … ask them to be adults about it…. if they had an injury…report to the physiotherapist… the player has to take responsibility…keep (their) side of it’. Furthermore, Chartered Physiotherapist Cory reported that, ‘having a management team that’s very like we support you wholeheartedly if…you want to access physiotherapist or S&C……girls (need) to know they won’t be punished …be dropped from the panel because … they’ve reported … important that they feel supported and cared for… the management set the tone really early on’. Terry stated, ‘honesty between everybody, honesty between the management, honesty between the player, honesty in the physiotherapist… trust… honesty… communication … opening up to everyone’, while sports psychologist Robin described how, ‘everybody needs to be working together to know what’s going on with each player’. Ladies Gaelic football manager Billie further emphasised the importance of team-work stating, ‘I’m not roaring and balling at the physiotherapist... I have to have faith in my physiotherapist for my players to have faith in the physiotherapist, the manager has to have faith in the physiotherapist and has to really … emphasise that’.

It was stated that a team culture where injured players still feel part of the team can facilitate reporting. Managers Jamie and Shay respectively outlined that, ’… if players feel that they’re still being included in training…they’re not actually missing out…you are minding their injuries … if you have that kind of environment…players would be far more open with actually reporting’ and ‘just because you’re injured doesn’t mean you’ve turned into a bad player… we will support you…to get back as quickly as you can …injured or not…you’re on our team’. Athletic Therapist Eddie described how, ‘a clear pathway to return … a collaborative effort …just because you get an injury, you’re still on the team just in a different capacity… there’s ways back … (so) girls feel like they’re (still) part of the team…they’re not on their own’.

The quantitative and qualitative findings were largely consistent, particularly in highlighting the ASP’s perspective that elite ladies Gaelic games players do not report all injuries. There was broad consensus across findings that this was due to reasons including timing and nature of injury, fear of losing playing time or being side-lined and lack of immediate access to medical personnel; reporting was more likely if there were trusting relationships between players and ASP and a team culture where reporting was encouraged. Context was provided by the qualitative findings including the central role of managers in setting the reporting culture and of education and resources to underpin an open and honest reporting culture, to support players and ASP to manage injury and injury reporting.

## Discussion

A mixed methods research design was used to examine the perspectives of ASP on the injury reporting practices of elite ladies Gaelic games players. Several key findings from the quantitative and qualitative data showed alignment, with the themes of the player, the system and the environment framing the qualitative findings. There was a recognition that players do not report all injuries, particularly when they considered an injury to be minor, could manage it themselves, or felt reporting may jeopardise their place on the team. A large majority of participants agreed that players did not report injuries due to a fear of losing playing time, being side-lined or letting the team down. More experienced players were considered more likely to report injuries compared to younger players. Players were more likely to report if they had immediate access to team medical personnel, had built trusting relationships with the team’s ASP and if there was a clear reporting pathway. Largely, managers were viewed as supportive of injured players, but it was also acknowledged that inconsistent or adverse reactions to player injury did arise.

A key finding in this study was the perceived difference in confidence between experienced and younger players to report injuries, a disparity reported and outlined previously in other female sporting cohorts. In elite female soccer, inexperienced players felt they did not have a voice in discussions with and between ASP, negatively impacting injury management [16]. Female intercounty players are younger on average and a high proportion are students [48]. Younger less-experienced players can be supported by the example of more seasoned players [36]. Research has indicated that peer mentored elite athletes competing in both individual and team-sports, showed enhanced performance and confidence through more constructive interactions with coaches, while mentors often served as a liaison between the athlete and the coach [37]. Mentoring relationships can be formal in nature or may develop organically [37]. The current state of play, and role of mentoring to support players and ASP in elite ladies Gaelic games, merits investigation.

Findings in this study highlight the competitiveness of the intercounty level where maintaining a place on the team may be prioritised over health. Findings also corroborate the research on reporting behaviours in elite female athletes [8,49], that players try to conceal injury by attending a clinician not affiliated with the team or perceiving they can manage the injury themselves. The commitment and sacrifice required to achieve sporting excellence can shape individuals’ athletic identity [30]. This can be abruptly lost because of injury, thus the willingness to play on when injured [50], or to delay injury reporting and assessment. The threat to athletic identity due to injury has been reported in elite adolescent athletes and elite male Gaelic footballers and hurlers [30,51]. Loss of athletic identity can impact players’ sense of worth with emotional, psychological and social consequences [30]. Therefore, ASP and players need to understand the nature of the athletic identity of elite ladies Gaelic games players’ particularly when they are navigating disappointments such as injury [1]. A greater appreciation of athletic identity in the sociocultural context of elite ladies Gaelic games is needed to optimally support the health and wellbeing of these players.

Players can be supported through regular ASP meetings and dialogue, offering an opportunity for injury, performance and wellbeing concerns to be discussed without fear of repercussions. The format can depend on what is considered contextually feasible and agreed by all stakeholders. These meetings could be conceptualised as occurring across two distinct formats- informal and formal. Informal interactions are characterised by the routine visibility and availability of ASP during training sessions, enabling spontaneous and context-driven discussions between ASP and players. These informal contacts should be ongoing and embedded within weekly operations. The formal meetings by contrast can involve more structured engagement. For these formal ASP and player meetings, the frequency remains context dependent, however a minimum of fortnightly is recommended to ensure continuity but with flexibility to increase frequency during periods of heightened injury risk or when managing complex cases.

By witnessing trusting relationships and open honest communication, players can be encouraged to be more open to report. As exposure to established behaviours and culture can make them more acceptable [52], structured communication pathways within the LGF and camogie communities can facilitate effective communication [53].

The importance of education to enhance injury was highlighted in the findings of this study. A lack of clinical understanding of injuries by management has been reported as a barrier to reporting by elite ladies Gaelic games players and field hockey players [8,49]. ASP in this study considered players more likely to report with a clear reporting pathway in place. In comparison, elite ladies Gaelic games players placed less value on having clear reporting pathways [8]. Yet reporting of injury is essential for its effective management and to keep players performing [36]. However, possible gaps in the education of players and ASP, including understanding of injury and its management, need to be identified and addressed [16]. In LGF, coaches and players have called for greater context-specific education and guidance on injury and prevention, with governing body support to ensure implementation [53,54]. There is a need therefore to prioritise educational programmes in the format preferred by individual stakeholders [3], with regular reviews and updates [55] based on research, dialogue with stakeholders and surveillance informed data.

Consistent access to ASP – specifically physiotherapists – was reported to encourage injury reporting. Insufficient funding for medical care and lack of availability of medical personnel has been highlighted in elite female soccer, with unavailability associated with playing with injury [16]. Comparatively, injury rates decreased while internal communication and strategies to prevent injuries improved in elite male soccer with improved access to health professionals [56]. Ladies are less well supported in Gaelic games compared to their male counterparts with significantly different budget profiles across the male and female organisations [57]. A small proportion of participants agreed that elite male Gaelic games players are more likely to report compared to females. This may be reflective of the inequity in expertise and resources that elite female players have access to relative to males [58]. Therefore, consistent and regular access to ASP, specifically physiotherapists, that players know and trust, can encourage injury reporting [25].

The Gaelic games player pathway has been developed to prioritise and support players across all levels of play [6]. A team charter for adult ladies Gaelic intercounty teams, including minimum standards for provisions of medical care, was agreed from the 2024 season onwards [48,59]. However, resource constraints can make it difficult for some counties to align to these agreed minimum standards [48]. Integration between the three Gaelic games organisations, to be implemented by 2027, recognises the vital role of women in sport with a goal to support women and men equally [57,60]. The development of this new Gaelic games organisation provides an opportunity for change including new culture and systems for participants [57]. However, despite these recent developments and recommendations, the lack of, and importance of access to ASP and resources in elite ladies Gaelic games has been highlighted [8].

Trusting relationships were identified as necessary to encourage injury reporting and for successful injury management. In addition to consistent access, participants considered the competence and qualifications of ASP as influential in building this trust. In elite male and female soccer, the interpersonal skills of the ASP were considered highly important in fostering trusting relationships [16,36]. The importance of interpersonal skills in Gaelic games, to maximise effective communication, relationship building, player development and team success is being highlighted, with resources made available through the Gaelic games’ associations [61–63].

Therefore, as a foundation to build a successful and cohesive team, it is essential that teams provide education to all stakeholders on the value and importance of injury reporting and potential risks that playing with injury poses to both player health and well-being and team performance. Concurrent to this is the necessity for improved access to medical personnel to ensure timely reporting and management of injury and return to play.

While ASP in this study largely disagreed that managers and teammates perceive injured players as ‘weak’, they did admit that managers may become annoyed or irritated when players are injured. This contrasts with the perspectives of elite ladies Gaelic games players who reportedly conceal injury due to a fear of being judged [8]. They further perceive a climate where injury reporting is viewed negatively, and report a fear of losing favour with management. This emphasises the vulnerability of players who depend on management for selection, as well as support and encouragement from their teammates [64]. It is of upmost importance to establish an interpersonal environment characterised by trust, respect, support and appreciation of players and ASP [52]. To facilitate this, Interpersonal skills training needs to be embedded into the educational programme for stakeholders and integrated into policy and practice.

This current study reported that team culture is shaped by contributions from all stakeholders with an atmosphere of openness and shared decision making integral to building trusting relationships. In elite soccer and competitive rowing, athletes were more likely to report injuries in a culture that allowed them to be open and honest [36,64]. Establishing this culture and environment in elite ladies Gaelic games requires a system change with active involvement from all stakeholders who have a shared goal of optimising individual and team performance.

### Limitations

While this study does provide a useful insight into perspectives of ASP on injury reporting in elite ladies Gaelic games, it does have some limitations. Due to the unique Irish cultural context of Gaelic games, findings may have limited generalisability to other sports. While face validity was established for the survey instrument, reliability was not, which may impact replicability of the study. Those who participated in this study have provided valuable and meaningful contributions from a multidisciplinary perspective. However, the sample did not include ASP from all domains or from all intercounty teams, while camogie was less represented. This underrepresentation of certain ASP roles, geographical regions and sports must therefore be considered when interpreting the results. Those who participated may overrepresent those ASP who had a greater interest in injury and injury reporting and while they have provided a highly valuable perspective, the thoughts of ASP with differing views would be equally valuable [53]. Strategies proposed for future studies to address sampling bias include quota-based recruitment to increase diversity and underrepresentation of certain ASP roles, geographical regions and sport codes. This could involve working in partnership with the Gaelic games NGBs, offering valuable access to networks and resources. This partnership with the LGFA and the Camogie Association could go some way to address the underrepresentation of camogie in this research, which limits the generalisability of the findings across both sports particularly considering the differing policies, procedures and player charters between the two NGBs. Qualitative research has the unique ability to capture human experiences and perspectives, however it is susceptive to subjective influences [65]. Nonetheless, the continual discussion and critical examination of the authors’ differing perspectives during the data collection and analysis, can strengthen confidence in the qualitative findings of this study [53].

## Conclusion

This study provides insights into the perspectives of ASP on injury reporting in elite ladies Gaelic games and acknowledges that players do not report all injuries. This is influenced by the interaction of socioecological factors including individual player factors, system-driven influences and environmental conditions. It is of critical importance that teams and organisation take measures to address this underreporting of injuries. These steps may include education for players and ASP on the importance and value of injury reporting to support early and appropriate intervention. Alongside this there is a need to put in place consistent and accessible medical resources to support elite ladies Gaelic games players. This would facilitate a team culture that priorities individual well-being in harmony with competitive success. Further prospective longitudinal injury tracking research should focus more attention on the teams’ system-driven influences and environmental conditions and how this impacts the reality of what does happen in response to injury within elite ladies Gaelic games.

## Supporting information

S1 File Survey instrument. (TIF)

S2 File Semi-structured interview guide. (TIF)

S1 Table Standards for reporting qualitative research checklist. (TIF)

S2 Table Survey responses. (TIF)
