# Supplementary figures and images for: Injury reporting in elite ladies Gaelic football and camogie: Perspectives of athlete support personnel
Source: PLoS One. 2025 Aug 4;20(8):e0329679. doi: 10.1371/journal.pone.0329679 (PMC12321058; doi:10.1371/journal.pone.0329679)

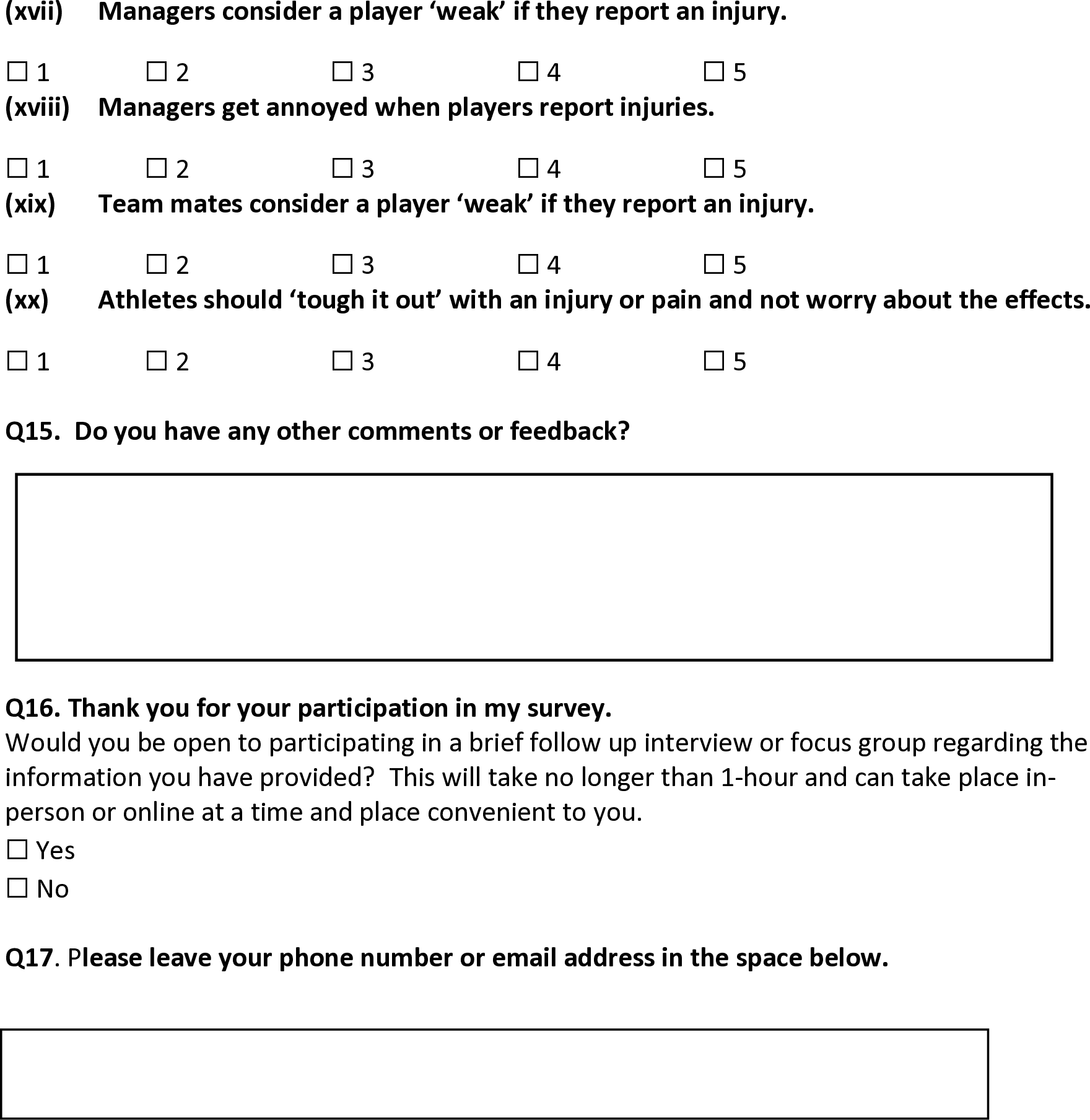

Supplement: S1 File — (TIF) [file pone.0329679.s001.tif]

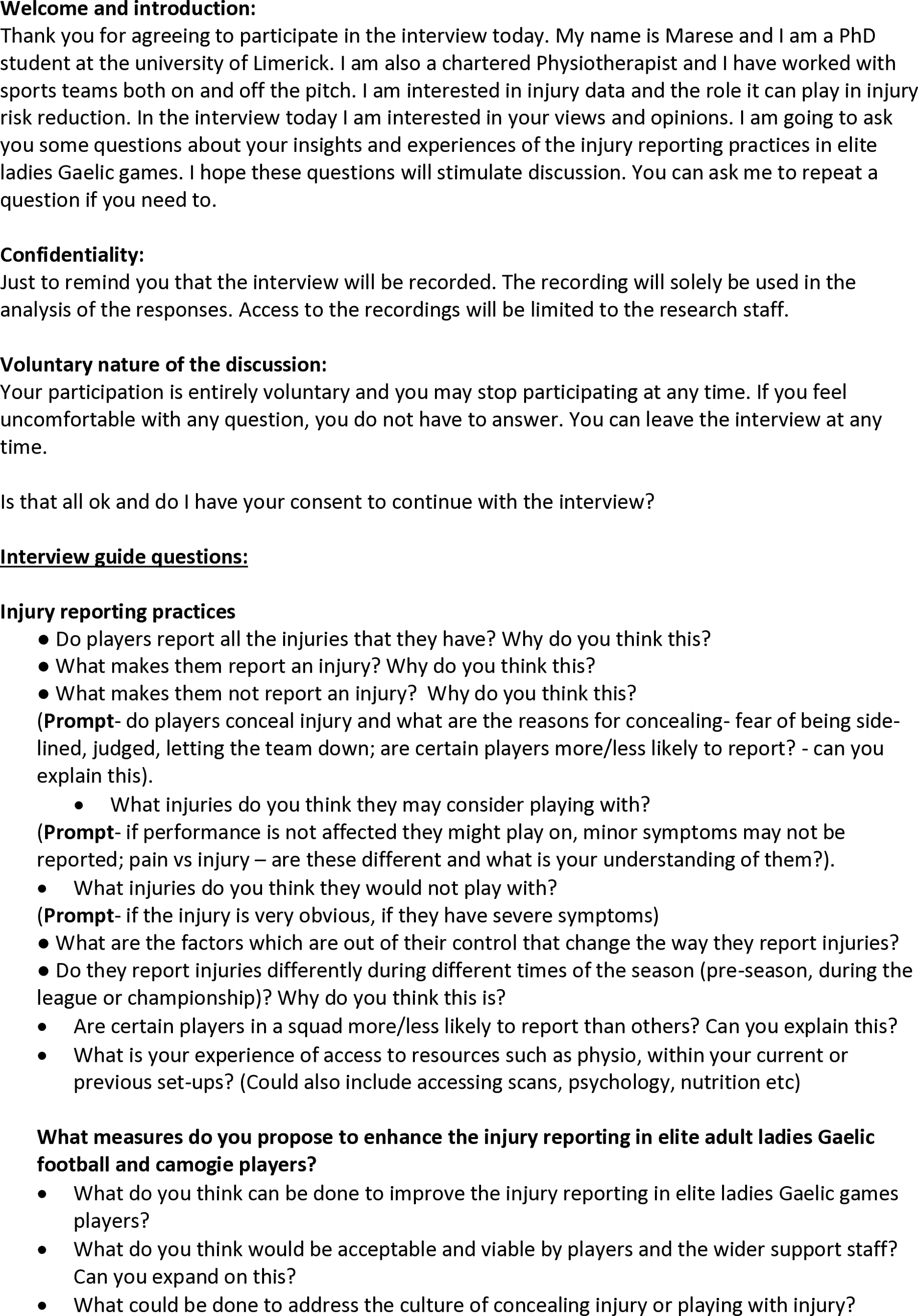

Supplement: S2 File — (TIF) [file pone.0329679.s002.tif]

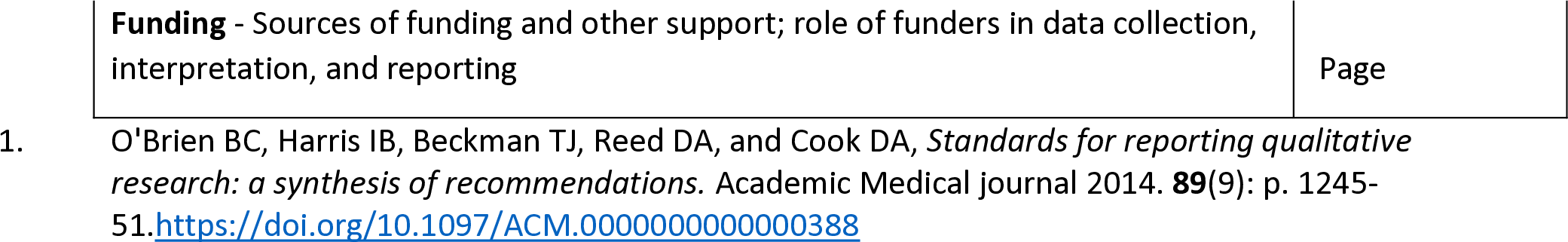

Supplement: S1 Table — (TIF) [file pone.0329679.s003.tif]

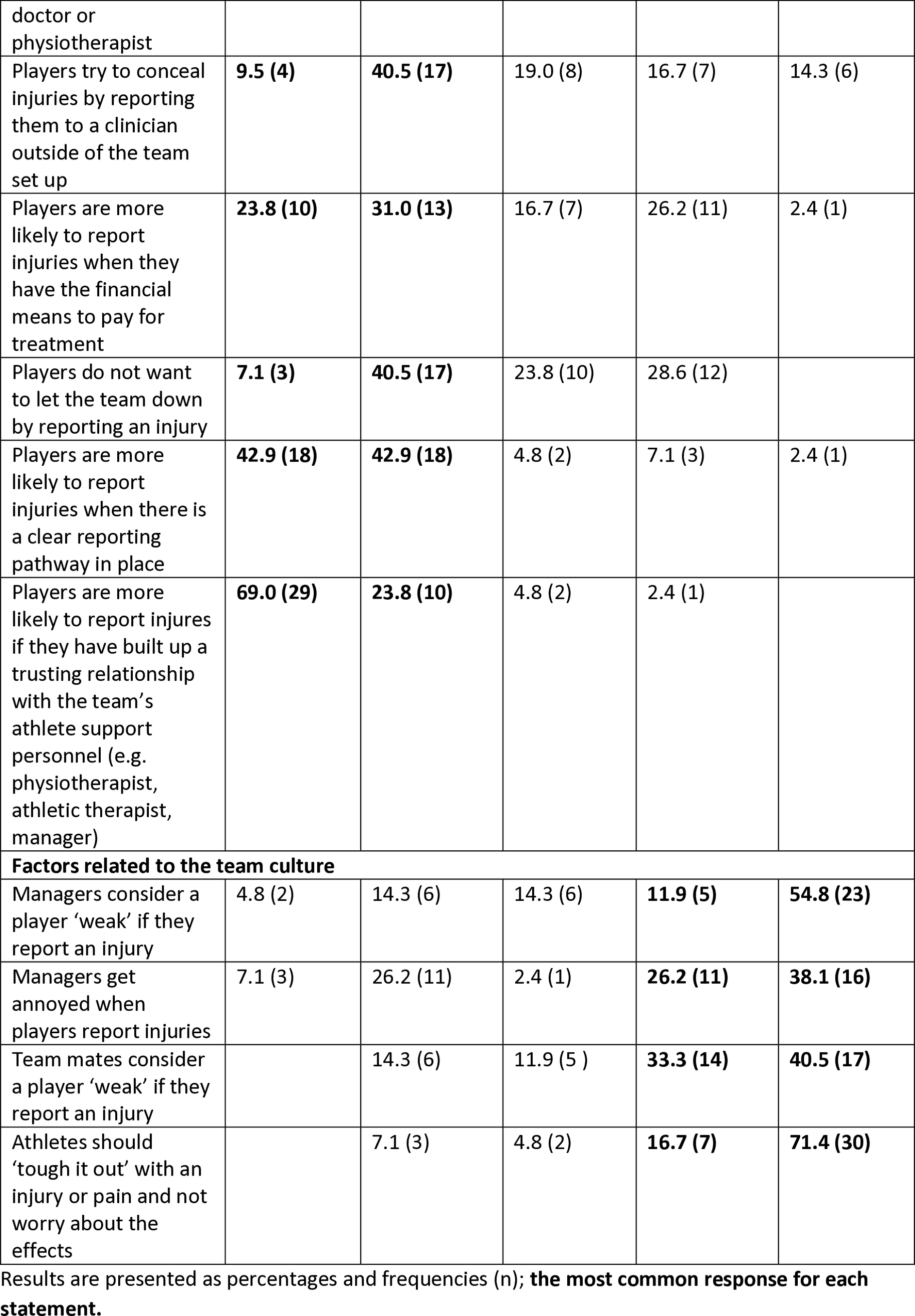

Supplement: S2 Table — (TIF) [file pone.0329679.s004.tif]
